# Supplementary material for: Local information sources received the most attention from Puerto Ricans during the aftermath of Hurricane Maria
Source: PLoS One. 2021 Jun 9;16(6):e0251704. doi: 10.1371/journal.pone.0251704 (PMC8189509; doi:10.1371/journal.pone.0251704)
Supplement: S1 Fig — (PDF) [file pone.0251704.s001.pdf]

S1 Fig.

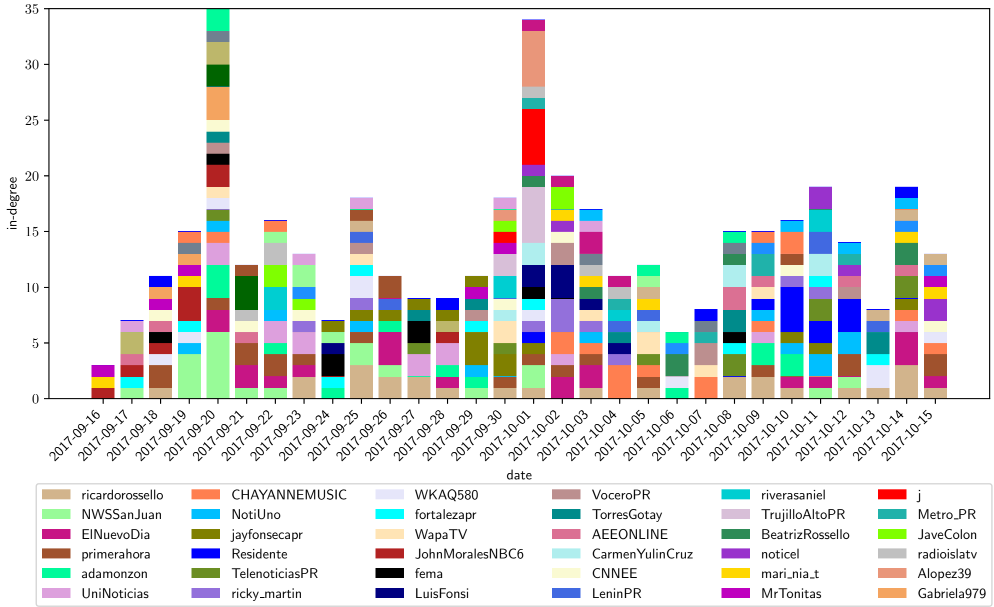

Stacked bar chart time-series showing the daily in-degrees of nodes in the top-fifty by in-degree of the aggregate network. Here, the individual nodes are broken out of their categories shown in Fig 11 and labeled separately to show individual contribution.
